# Supplementary material for: Duodenal bacterial proteolytic activity determines sensitivity to dietary antigen through protease-activated receptor-2
Source: Nat Commun. 2019 Mar 13;10:1198. doi: 10.1038/s41467-019-09037-9 (PMC6416356; doi:10.1038/s41467-019-09037-9)
Supplement: Supplementary file 1 — Supplementary Information [file 41467_2019_9037_MOESM1_ESM.pdf]

Supplemental Information for:

Duodenal bacterial proteolytic activity determines sensitivity to dietary antigen through protease-activated receptor-2

Caminero *et al*

This PDF includes:

Supplementary Tables 1-4

Supplementary Figures 1-8

Supplementary Tables

Diagnosis	Average age	Sex (% of females)
Controls	47 (30-72)	75 %
Celiac disease	42.3 (18-64)	61.5 %

Supplementary Table 1. Demographics of controls and celiac disease patients recruited for duodenal biopsies and aspirates.

Strain or vector	Relevant characteristic- genotype	Source
**Strain**		
Pseudomonas aeruginosa PA14	Laboratory wild-type strain; Burn patient isolate.	1
Pseudomonas aeruginosa PA14 *lasB::MAR2xT7*	Transposon mutant in *lasB* gene.	1
Pseudomonas aeruginosa PA14 p*lasB-luxCDABE*	PA14 with integrated p*lasB-luxCDABE* transcriptional reporter at the CTX integration site.	This study
Pseudomonas aeruginosa X46.1	Clinical isolate from the small intestine of a celiac disease patient.	2
NEB 5-alpha competent *Escherichia coli*	Chemically competent *E. coli* strain suitable for high efficiency transformation. Derivative of the *E.coli* DH5α strain.	New England Biolabs
Escherichial coli HB101	Competent *E.coli* strains. genotype: F- mcrB mrr hsdS20(rB - mB -) recA13 leuB6 ara-14 proA2 lacY1 galK2 xyl-5 mtl-1 rpsL20(SmR) glnV44 λ	Lab collection
**Vector**		
pCDS108	Promoter of *lasB* fused to *luxCDABE* (p*lasB-luxCDABE)* on a vector suitable for genome integration at the CTX site	3
pRK2013	Mobilizing vector, ColE1 Tra (RK2); Km^R	4
pHERD26T	Broad-host range shuttle vector, Tet^R	5
pHERD26T-lasBEnd	pHERD26T with 2.1 bp fragment containing *lasB* and its native promoter.	This study
pHERD26T-lasBAra	pHERD26T with 2.1 bp fragment containing *lasB* under the control of the arabinose inducible promoter.	This study
Nluc-hPAR2-eYFP	Detection of N-terminal PAR-2 cleavage. NanoLuc luciferase reporter tag was cloned in frame with the human PAR2 cDNA and its stop codon was mutated to insert eYFP tag.	6

Tet, tetracycline; Kanamycin, Km

Supplementary Table 2. Bacterial strains and vectors used in the study.

Primer	Sequence	Function	Origin
lasB F-end	5'-GTCGACTCTAGAGGATCCCCTGGCCCCTCGCTGAGCGC-3'	Cloning of LasB	Integrated DNA Technologies
lasB R-end	5'-AGAATTCGAGCTCGGTACCCCTGGCGGAAGACGGCTTGAGC-3'	Cloning of LasB	Integrated DNA Technologies
lasB F-ara	5'-AGAATTCGAGCTCGGTACCCCAGGAGAACTGAACAAGATGAAGAAGG-3'	Cloning of LasB	Integrated DNA Technologies
lasBR-ara	5'-GTCGACTCTAGAGGATCCCCTTACAACGCGCTCGGGCA-3'	Cloning of LasB	Integrated DNA Technologies
pHERD26TgF	5'-GGGGATCCTCTAGAGTCGAC-3'	Amplification of pHERD26T vector backbone for cloning LasB	Integrated DNA Technologies
pHERD26TgR	5'-GGGTACCGAGCTCGAATTCTTATCAGATC-3'	Amplification of pHERD26T vector backbone for cloning LasB	Integrated DNA Technologies
EUB338	5'-GCTGCCTCCCGTAGGAGT-3' (5′ end-labeled with Cy3)	Detection of bacterial DNA by 16S-FISH technique	Integrated DNA Technologies
341F	5'-CCTACGGGAGGCAGCAG-3'	Microbiota sequencing for Ilumina	Life Technologies
518R	5'-GTATTACCGCGGCTGCTGG-3'	Microbiota sequencing for Ilumina	Life Technologies

Supplementary Table 3: Primers used in the study.

Antibody	Function	Dilution	Origin
Rabbit anti-human antibody to CD3	Primary antibody for staining of CD3 by IHC	1:2000	Dako- GA50361-2
HRP–conjugated anti-mouse IgG	Secondary antibody for anti-gliadin antibody determination	1:2000	GE Healthcare- NA931
HRP–conjugated anti-mouse IgA	Secondary antibody for anti-gliadin antibody determination	1:2000	Abcam- ab97235
Rabbit polyclonal antibody PAR-2 (H-99)	Primary antibody for detection of PAR-2 by IHC	1:500	Santa Cruz Biotechnology sc-57797
Alexa Fluor 594 goat anti-rabbit IgG	Secondary antibody	1:2000	Life Technologies A11037
CD45-BV421 (30-F11)	Fluorochrome-labeled cell-surface antibody for cytometry analysis.	1:100	BioLegend-103133
CD3ε-PE-dazzle 594 (145-2C11)	Fluorochrome-labeled cell-surface antibody for flow cytometry analysis.	1:100	BioLegend-100348
CD103-PE (3E7)	Fluorochrome-labeled cell-surface antibody for flow cytometry analysis.	1:100	BioLegend-121405

HRP, horseradish peroxidase; IHC, immunohistochemistry

Supplementary Table 4: Antibodies used in the study.

Supplementary Figures

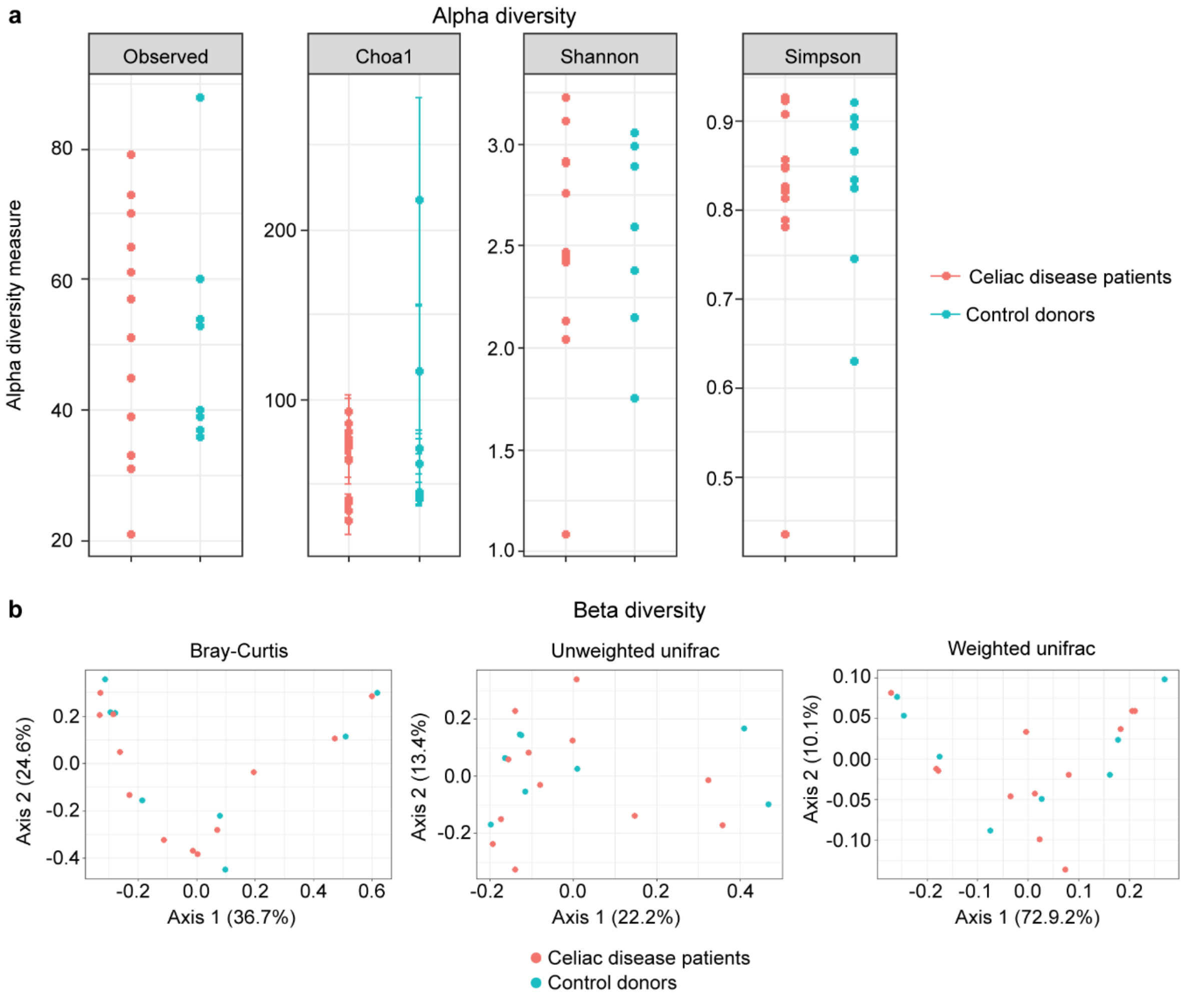

Supplementary Figure 1. No difference in alpha or beta diversity in CeD and control donors**. a.** Alpha-diversity of microbiota profiles in duodenal biopsies from patients with CeD (n=12) and without CeD (n=8) measured as Observed species, Chao1, Shannon, and Simpson index. **b.** Beta-diversity of microbiota profiles from duodenal biopsies of patients with CeD (n=12) and without CeD (n=8), using Bray-Curtis dissimilarity and Unifrac (weighted and unweighted) represented as principal coordinates. Each dot represents an individual human donor.

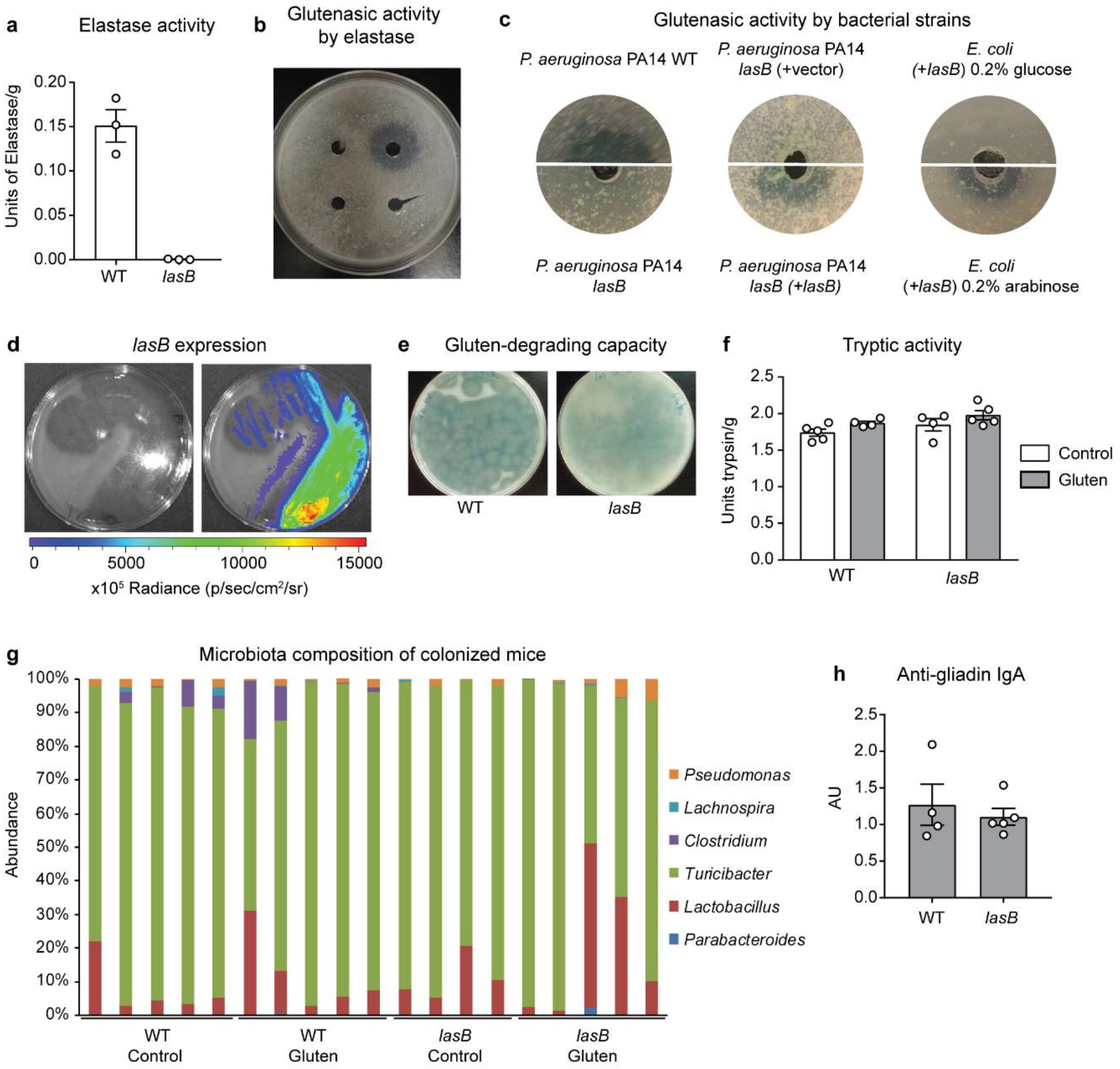

Supplementary Figure 2. LasB is expressed *in vivo* and degrades gluten. **a.** Elastase activity of *P. aeruginosa* PA14 WT (n=3) and its *lasB* mutant (n=3). Data is presented as mean ± s.e.m where each dot represents an individual biological replicate. **b.** Glutenasic activity of commercial LasB elastase. One representative bioassay is shown from 3 independent experiments. **c.** Glutenasic activity measured in different bacterial strains with and without lasB gene expression. *P. aeruginosa* PA14 WT and its mutant *lasB* are shown in the left panel. *P. aeruginosa* PA14 *lasB* with empty vector *pHERD26T* (+vector) or with vector *pHERD26T* complemented with *lasB* under the *P. aeruginosa* PA14 *lasB* endogenous promoter (+*lasB*) are shown in the center panel. NEB 5-alpha competent *E. coli* strain with vector *pHERD26T* complemented with *lasB* under the arabinose promoter, repressed by the addition of glucose (+*lasB* 0.2% glucose) or induced by the addition of arabinose (+*lasB* 0.2% arabinose) are shown in the right panel. Representative bioassays are shown from 3 independent experiments. **d.** Luminescence linked to expression of *lasB* corresponding with degradation of gluten by *P. aeruginosa* PA14 harboring a *lasB-luxCDABE* transcriptional reporter at the CTX integration site. One representative image is shown from 3 biological replicates. **e.** Degradation

of gluten by *P. aeruginosa* PA14 WT and the *lasB* mutant strains *in vitro* recovered from the small intestine of clean SPF C57BL/6 mice. Representative images are shown from 5 mice. **f.** Tryptic activity measured in the small intestine of clean SPF C57BL/6 mice colonized with *P. aeruginosa* PA14 WT or the *lasB* mutant*,* treated with gluten (grey bars; n=4 WT, n=4 lasB) or without gluten (controls, white bars; n=5 WT, n=4 lasB)*.* Data presented as mean ± s.e.m. where each dot represents an individual mouse. **g.** Small intestinal microbiota composition, expressed as relative abundance, at the genus level in clean-SPF C57BL/6 mice colonized with *P. aeruginosa* PA14 WT or the *lasB* mutant treated with gluten (n=5 WT, n=5 lasB) or without gluten (controls; n=5 WT, n=4 lasB). **h.** Anti-gliadin IgA antibody titers in small intestinal washes of clean-SPF C57BL/6 mice colonized with *P. aeruginosa* PA14 WT or the *lasB* mutant and treated with gluten (n=4 WT, n=4 *lasB*). Data presented as mean ± s.e.m. where each dot represents an individual mouse.

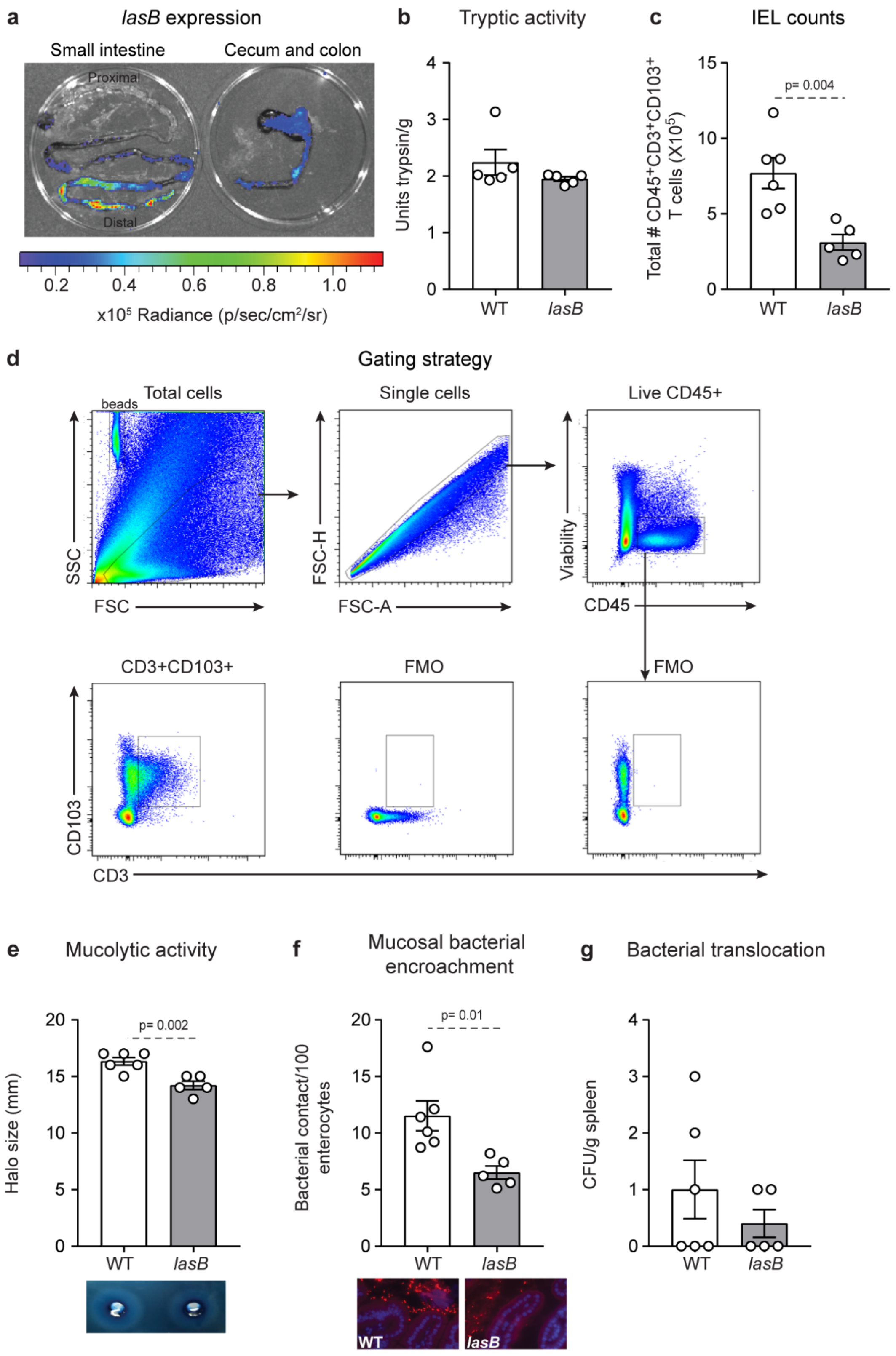
a
lasB expression
Small intestine
Cecum and colon
Proximal
Distal
0.2 0.4 0.6 0.8 1.0
x10⁵ Radiance (p/sec/cm²/sr)
b
Tryptic activity
Units trypsin/g
WT lasB
c
IEL counts
Total # CD45+CD3+CD103+ T cells (X10⁵)
p= 0.004
WT lasB
d
Gating strategy
Total cells
beads
SSC
FSC
Single cells
FSC-H
FSC-A
Live CD45+
Viability
CD45
CD3+CD103+
FMO
FMO
CD103
CD3
e
Mucolytic activity
Halo size (mm)
p= 0.002
WT lasB
f
Mucosal bacterial encroachment
Bacterial contact/100 enterocytes
p= 0.01
WT lasB
WT
lasB
g
Bacterial translocation
CFU/g spleen
WT lasB

Supplementary Figure 3. LasB induces a pro-inflammatory response *in vivo* in the absence of microbiota. **a.** Visual expression of *lasB* in the small intestine (left panel) and large intestine (right panel) of C57BL/6 mice monocolonized with *P. aeruginosa* PA14 harboring a *lasB-luxCDABE* transcriptional reporter, which allows direct monitoring of the *lasB* promoter activity via the detection of light (n=3). Representative images are shown. **b.** Tryptic activity measured in the small intestine of ex-germ free C57BL/6 mice monocolonized with *P. aeruginosa* PA14 WT (n=5) or the *lasB* mutant (n=5). **c.** Total number of CD45+CD3+CD103+ cell population in the isolated small intestinal intraepithelial lymphocyte (IEL) compartment in C57BL/6 mice monocolonized with *P. aeruginosa* PA14 WT (n=6) or the *lasB* mutant (n=5). **d**. Gating strategy for determination of CD45+CD3+CD103+ IELs. CD3+CD103+ cells were determined from single cells gated on live CD45+ cells. Total cell counts were determined using counting beads. **e.** Luminal small intestinal mucolytic activity in mice monocolonized with *P. aeruginosa* PA14 WT (n=6) or the *lasB* mutant (n=5). Representative bioassays are shown. **f.** Quantitative measurement of bacterial-epithelial contact, by 16S-fluorescence *in situ* hybridization (FISH), in the small intestinal mucosa of mice monocolonized with *P. aeruginosa* PA14 WT (n=6) or the *lasB* mutant (n=5). Representative stained small intestinal sections are shown. **g.** Translocation of live bacteria to the spleen in C57BL/6 mice monocolonized with *P. aeruginosa* PA14 WT (n=6) or the *lasB* mutant (n=5). In panels b, c, e and f data presented as mean ± s.e.m. where each dot represents an individual mouse. Displayed p vales calculated by Student's t-test.

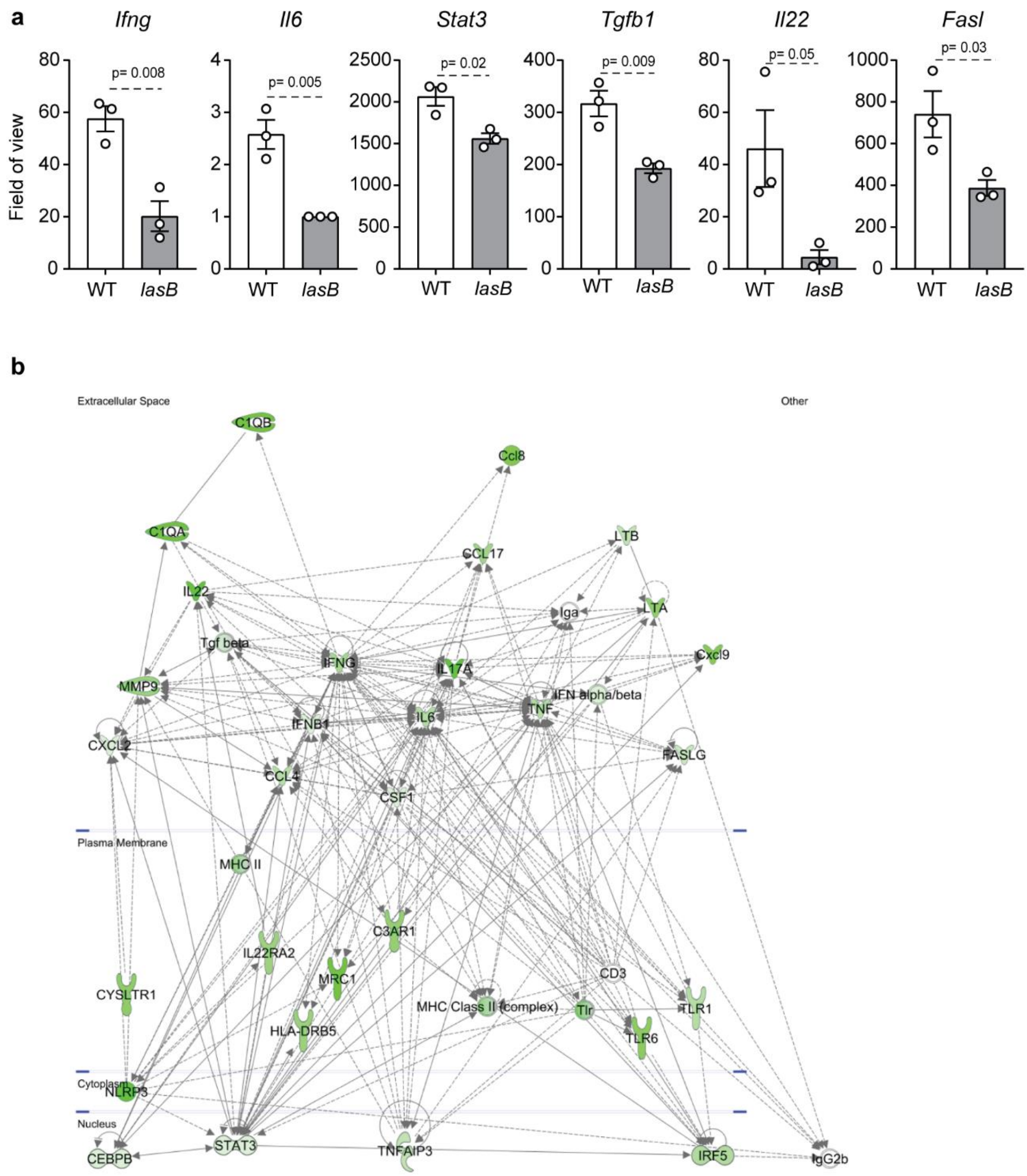

Supplementary Figure 4. LasB induces a pro-inflammatory gene signature in the IEL compartment. **a.** Gene expression in the IEL compartment of the small intestine of mice colonized with *P. aeruginosa* PA14 WT (n=3) or the *lasB* mutant (n=3), assessed by NanoString nCounter gene expression. Data presented as mean ± s.e.m. where each dot represents an individual mouse. Displayed p vales calculated by Student's t-test. **b.** Canonical pathways dysregulated in C57BL/6 mice monocolonized with *P. aeruginosa* PA14 WT or the *lasB* mutant, generated by Ingenuity Pathway Analysis (IPA) software based on downregulated (green) genes in the IEL compartment of *lasB*-colonized mice.

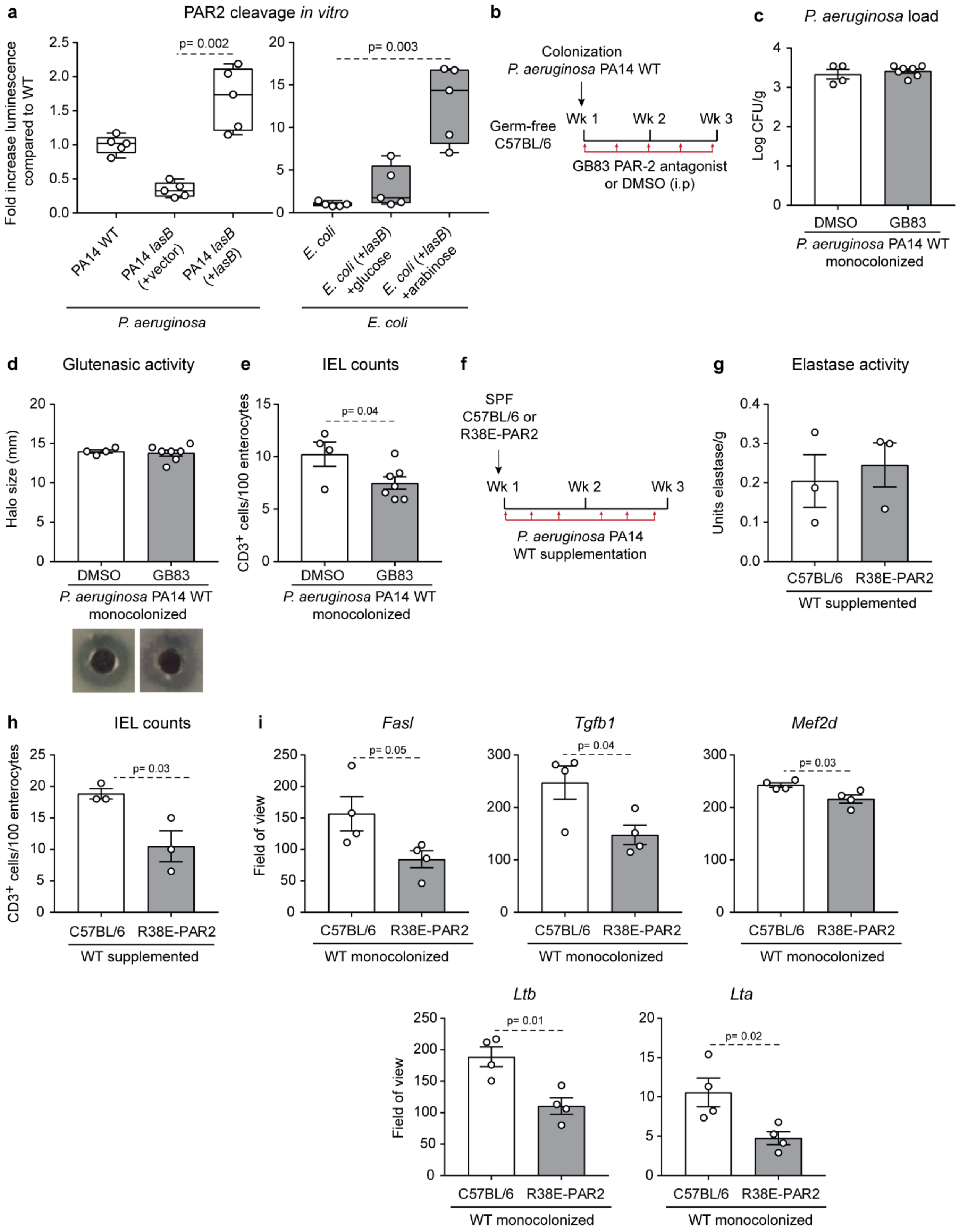
a
PAR2 cleavage in vitro
Fold increase luminescence compared to WT
p= 0.002
p= 0.003
PA14 WT
PA14 lasB (+vector)
PA14 lasB (+lasB)
P. aeruginosa
E. coli
E. coli (+lasB) +glucose
E. coli (+lasB) +arabinose
E. coli
b
Colonization
P. aeruginosa PA14 WT
Germ-free C57BL/6
Wk 1
Wk 2
Wk 3
GB83 PAR-2 antagonist or DMSO (i.p)
c
P. aeruginosa load
Log CFU/g
DMSO
GB83
P. aeruginosa PA14 WT monocolonized
d
Glutenasic activity
Halo size (mm)
DMSO
GB83
P. aeruginosa PA14 WT monocolonized
e
IEL counts
CD3+ cells/100 enterocytes
p= 0.04
DMSO
GB83
P. aeruginosa PA14 WT monocolonized
f
SPF C57BL/6 or R38E-PAR2
Wk 1
Wk 2
Wk 3
P. aeruginosa PA14 WT supplementation
g
Elastase activity
Units elastase/g
C57BL/6
R38E-PAR2
WT supplemented
h
IEL counts
CD3+ cells/100 enterocytes
p= 0.03
C57BL/6
R38E-PAR2
WT supplemented
i
Fasl
Field of view
p= 0.05
C57BL/6
R38E-PAR2
WT monocolonized
Tgfb1
p= 0.04
C57BL/6
R38E-PAR2
WT monocolonized
Mef2d
p= 0.03
C57BL/6
R38E-PAR2
WT monocolonized
Ltb
Field of view
p= 0.01
C57BL/6
R38E-PAR2
WT monocolonized
Lta
p= 0.02
C57BL/6
R38E-PAR2
WT monocolonized

Supplementary Figure 5. LasB induces a pro-inflammatory response through PAR-2. **a.** *In vitro* cleavage of the external domain of PAR-2 by *P. aeruginosa* PA14 WT, *P. aeruginosa lasB*, *P. aeruginosa lasB* mutants complemented with an empty *pHERD26T* plasmid (+vector) or with the complemented *lasB*-expressing *pHERD26T* plasmid (+*lasB*) and NEB 5-alpha competent *E. coli* strains with vector *pHERD26T* complemented with *lasB* under the arabinose promoter, repressed by the addition of glucose (+glucose) or induced by the addition of arabinose (+arabinose) (n=5/group). Data presented as median with interquartile range and whiskers extending from minimum to maximum, which each dot representing a biological replicate. Displayed p values calculated by Kruskal-Wallis with Dunn's post-hoc test. Results are shown as fold increase of luminescence intensity obtained after PAR2 cleavage compared to *P. aeruginosa* PA14 WT or *E. coli* WT. **b.** Protocol for treatment of *P. aeruginosa* PA14 WT-monocolonized mice with GB83 PAR-2 antagonist or DMSO. **c.** *P. aeruginosa* bacterial load in the small intestine of C57BL/6 mice monocolonized with *P. aeruginosa* PA14 WT treated with GB83 PAR-2 antagonist (n=7) or DMSO (n=4). **d.** Small intestinal glutenasic activity from C57BL/6 mice monocolonized with *P. aeruginosa* PA14 WT treated with GB83 PAR-2 antagonist (n=7) or DMSO (n=4). Representative bioassays are shown. **e.** Quantitative measure of IELs/100 enterocytes in small intestinal villi tips of C57BL/6 mice monocolonized with *P. aeruginosa* PA14 WT treated with GB83 PAR-2 antagonist (n=7) or DMSO (n=4). **f.** Protocol for the supplementation of SPF C57BL/6 and protease-resistant PAR-2 mutant mice (PAR38E-PAR2) with *P. aeruginosa* PA14 WT. **g.** Luminal small intestinal elastase activity from SPF C57BL/6 mice (n=3) and protease-resistant PAR-2 mutant mice (R38E-PAR2; n=3)supplemented with *P. aeruginosa* PA14 WT. **h.** Quantitative measure of IELs/100 enterocytes in small intestinal villi tips of SPF C57BL/6 mice (n=3) and R38E-PAR2 mice (n=3) supplemented with *P. aeruginosa* PA14 WT. **i.** Gene expression in the IEL compartment of the small intestine of ex-germ free C57BL/6 mice (n=4) and PAR38E-PAR2 mice (n=4) monocolonized with *P. aeruginosa* PA14 WT, assessed by NanoString nCounter gene expression. Data in panels c-e, g-i presented as mean ± s.e.m. where each dot represents an individual mouse. Displayed p values calculated by Student's t-test.

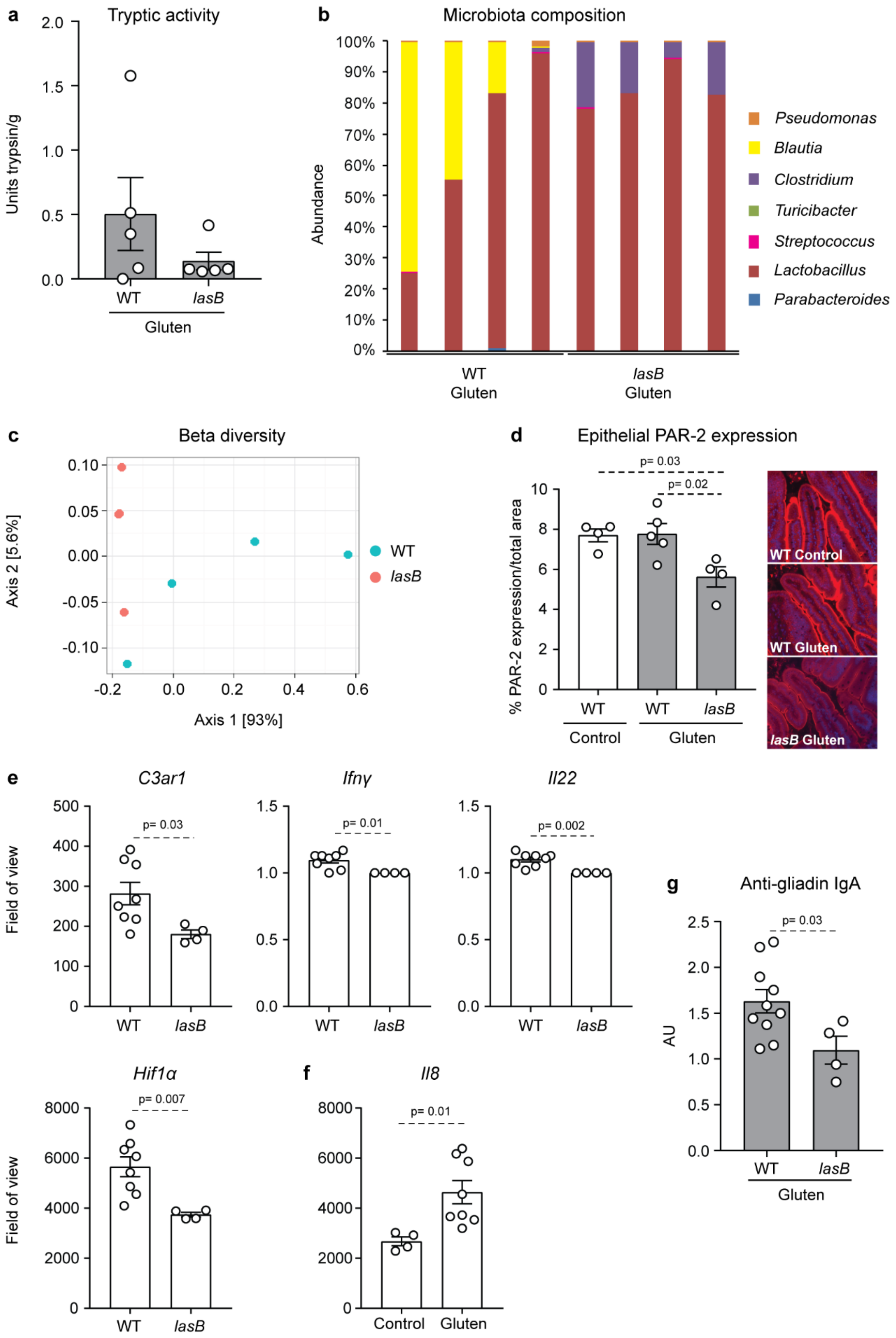
a
Tryptic activity
Units trypsin/g
2.0
1.5
1.0
0.5
0.0
WT
lasB
Gluten
b
Microbiota composition
Abundance
100%
90%
80%
70%
60%
50%
40%
30%
20%
10%
0%
Pseudomonas
Blautia
Clostridium
Turicibacter
Streptococcus
Lactobacillus
Parabacteroides
WT
Gluten
lasB
Gluten
c
Beta diversity
Axis 2 [5.6%]
0.10
0.05
0.00
-0.05
-0.10
-0.2
0.0
0.2
0.4
0.6
Axis 1 [93%]
WT
lasB
d
Epithelial PAR-2 expression
% PAR-2 expression/total area
p= 0.03
p= 0.02
10
8
6
4
2
0
WT
WT
lasB
Control
Gluten
WT Control
WT Gluten
lasB Gluten
e
C3ar1
Field of view
500
400
300
200
100
0
p= 0.03
WT
lasB
Ifnγ
1.5
1.0
0.5
0.0
p= 0.01
WT
lasB
Il22
1.5
1.0
0.5
0.0
p= 0.002
WT
lasB
g
Anti-gliadin IgA
AU
2.5
2.0
1.5
1.0
0.5
0.0
p= 0.03
WT
lasB
Gluten
Hif1α
Field of view
8000
6000
4000
2000
0
p= 0.007
WT
lasB
f
Il8
8000
6000
4000
2000
0
p= 0.01
Control
Gluten

Supplementary Figure 6. LasB enhances gluten-induced pathology in NOD/DQ8 mice through gluten-independent mechanisms. **a.** Tryptic activity measured in the small intestine of clean SPF NOD/DQ8 mice colonized with *P. aeruginosa* PA14 WT (n=5) or the *lasB* mutant (n=5). **b.** Small intestinal microbiota composition at the genus level in clean SPF NOD/DQ8 mice colonized with *P. aeruginosa* PA14 WT (n=4) or the *lasB* mutant (n=4) and treated with gluten. **c.** β-diversity Bray Curtis dissimilarity PCoA plot of microbiota profiles from clean SPF NOD/DQ8 mice colonized with *P. aeruginosa* PA14 WT (n=4) or the *lasB* mutant (n=4). **d.** Immunostaining of PAR-2 in the small intestine of clean SPF NOD/DQ8 mice colonized with *P. aeruginosa* PA14 WT or the *lasB* mutant treated with gluten (n=5 WT, n=4 *lasB*) or without gluten (control; n=4 WT). **e.** Gene expression in whole small intestinal tissue of NOD/DQ8 mice colonized with *P. aeruginosa* PA14 WT or the *lasB* mutant, when comparisons were performed between *P. aeruginosa* PA14 WT (n=8) and *lasB* (n=4) colonized mice, assessed by NanoString nCounter Gene Expression CodeSets. **f.** Gene expression in whole small intestinal tissue of NOD/DQ8 mice colonized with *P. aeruginosa* PA14 WT or the *lasB* mutant, when comparisons were performed between gluten treated (n=8) and control mice (n=4), assessed by NanoString nCounter Gene Expression CodeSets. **g.** Anti-gliadin IgA antibodies in small intestinal washes of clean SPF NOD/DQ8 colonized mice with *P. aeruginosa* PA14 WT (n=10) or the *lasB* mutant (n=4) and treated with gluten. Data in panels a, d, e-g presented as mean ± s.e.m where each dot represents an individual mouse. Displayed p values calculated by one-way ANOVA with a Tukey post-hoc test (d) or Student's t-test (a, e-g).

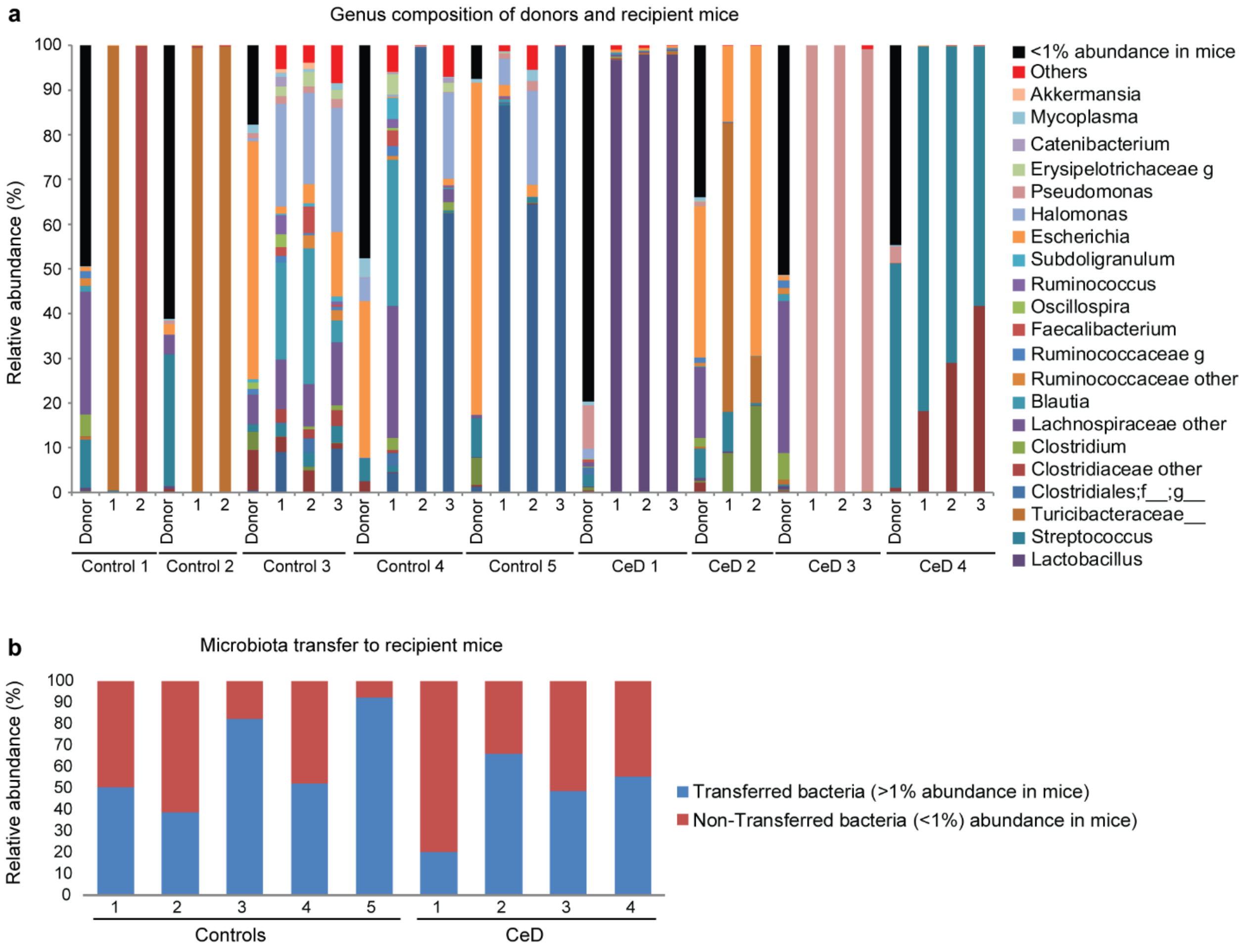

Supplementary Figure 7. Human small intestinal microbiota is transferred in ex-germ-free recipient mice. **a.** Microbial composition, at the genus level, of small intestinal contents from recipient mice colonized with human aspirates of patients with CeD (n=4 donors) and without CeD (controls; n=5 donors)) and from the corresponding duodenal biopsies of the donors used for mice colonization. Each donor was used to colonize 2-3 mice. Black bars represent those groups present in human duodenal biopsies not transferred to recipient mice and red bars represent those groups found at less than 1% of the relative abundance in mice. **b.** Percentage of microbiota transferred from the human aspirates of each donor to the small intestine of recipient mice.

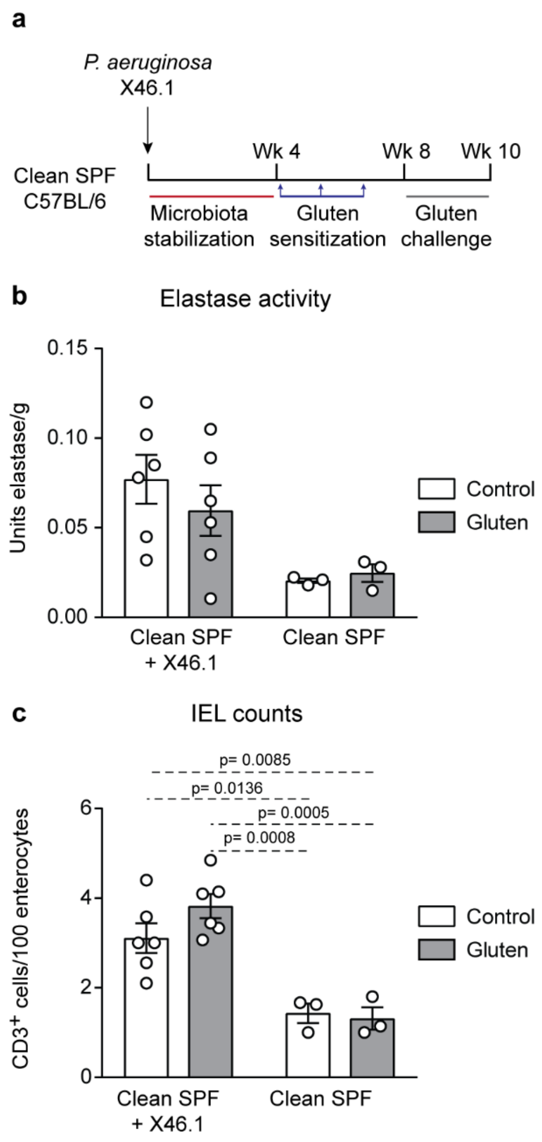

Supplementary Figure 8. A clinical *P. aeruginosa* strain isolated from the duodenum of a CeD patient enhances gluten-induced pathology in NOD/DQ8 mice**. a.** Protocol for gluten sensitization and challenge in clean SPF C57BL/6 mice colonized with *P. aeruginosa* X46.1. **b.** Elastase activity measured in the small intestine of clean SPF C57BL/6 mice colonized with *P. aeruginosa* X46.1 treated with gluten (grey bars; n=6 clean SPF + X46.1, n=3 clean SPF) or without gluten (white bars; n=6 clean SPF + X46.1, n=3 clean SPF). **c.** Quantitative measurement of IELs/100 enterocytes in small intestinal villi tips of clean SPF C57BL/6 colonized mice with *P. aeruginosa* X46.1*,* treated with gluten (grey bars; n=6 clean SPF + X46.1, n=3 clean SPF) or without gluten (white bars; n=6 clean SPF + X46.1, n=3 clean SPF). Data in panels b, c presented as mean ± s.e.m. where dots represent individual mice. Displayed p values calculated by one-way ANOVA with Tukey post-hoc test.

Supplementary References

1- Liberati NT*, et al.* An ordered, nonredundant library of Pseudomonas aeruginosa strain PA14 transposon insertion mutants. *Proc. Natl. Acad. Sci. U.S.A.* **103**, 2833-2838 (2006).

2- Herran AR*, et al.* Gluten-degrading bacteria are present in the human small intestine of healthy volunteers and celiac patients. *Res. Microbiol.* **168**, 673-684 (2017).

3- Sibley CD*, et al.* Discerning the complexity of community interactions using a Drosophila model of polymicrobial infections. *PLoS pathogens* **4**, e1000184 doi: 10.1371/journal.ppat.1000184 (2008).

4- Figurski DH, Helinski DR. Replication of an origin-containing derivative of plasmid RK2 dependent on a plasmid function provided in trans. *Proc. Natl. Acad. Sci. U.S.A.* **76**, 1648-1652 (1979).

5- Qiu D, Damron FH, Mima T, Schweizer HP, Yu HD. PBAD-based shuttle vectors for functional analysis of toxic and highly regulated genes in Pseudomonas and Burkholderia spp. and other bacteria. *Appl. Environ. Microbiol.* **74**, 7422-7426 (2008).

6- Mihara K*, et al.* Thrombin-Mediated Direct Activation of Proteinase-Activated Receptor-2: Another Target for Thrombin Signaling. *Mol. Pharmacol* **89**, 606-614 (2016).
.
