# Supplementary material for: Duodenal bacterial proteolytic activity determines sensitivity to dietary antigen through protease-activated receptor-2
Source: Nat Commun. 2019 Mar 13;10:1198. doi: 10.1038/s41467-019-09037-9 (PMC6416356; doi:10.1038/s41467-019-09037-9)
Supplement: Supplementary file 3 — Reporting Summary [file 41467_2019_9037_MOESM3_ESM.pdf]

nature research

Corresponding author(s): Elena F. Verdu

Last updated by author(s): Feb 7, 2019

Reporting Summary

Nature Research wishes to improve the reproducibility of the work that we publish. This form provides structure for consistency and transparency in reporting. For further information on Nature Research policies, see Authors & Referees and the Editorial Policy Checklist.

Statistics

For all statistical analyses, confirm that the following items are present in the figure legend, table legend, main text, or Methods section.

n/a	Confirmed	
☐	☒	The exact sample size (*n*) for each experimental group/condition, given as a discrete number and unit of measurement
☐	☒	A statement on whether measurements were taken from distinct samples or whether the same sample was measured repeatedly
☐	☒	The statistical test(s) used AND whether they are one- or two-sided<br>*Only common tests should be described solely by name; describe more complex techniques in the Methods section.*
☐	☒	A description of all covariates tested
☐	☒	A description of any assumptions or corrections, such as tests of normality and adjustment for multiple comparisons
☐	☒	A full description of the statistical parameters including central tendency (e.g. means) or other basic estimates (e.g. regression coefficient) AND variation (e.g. standard deviation) or associated estimates of uncertainty (e.g. confidence intervals)
☐	☒	For null hypothesis testing, the test statistic (e.g. *F*, *t*, *r*) with confidence intervals, effect sizes, degrees of freedom and *P* value noted<br>*Give P values as exact values whenever suitable.*
☒	☐	For Bayesian analysis, information on the choice of priors and Markov chain Monte Carlo settings
☐	☒	For hierarchical and complex designs, identification of the appropriate level for tests and full reporting of outcomes
☐	☒	Estimates of effect sizes (e.g. Cohen's *d*, Pearson's *r*), indicating how they were calculated

Our web collection on statistics for biologists contains articles on many of the points above.

Software and code

Policy information about availability of computer code

Data collection	NCBI database, Greengenes 2011 reference database
Data analysis	Cutadapt software (version 1.2.1), PANDAseq software (version 2.8), Qiime, sl1p pipeline, Nsolver 2.5 (NanoString Technologies) and Ingenuity Pathway Analysis software (Qiagen)

For manuscripts utilizing custom algorithms or software that are central to the research but not yet described in published literature, software must be made available to editors/reviewers. We strongly encourage code deposition in a community repository (e.g. GitHub). See the Nature Research guidelines for submitting code & software for further information.

Data

Policy information about availability of data

All manuscripts must include a data availability statement. This statement should provide the following information, where applicable:

- Accession codes, unique identifiers, or web links for publicly available datasets
- A list of figures that have associated raw data
- A description of any restrictions on data availability

Data is available in NCBI database, under the Umbrella BioProject: PRJNA518891. This project contains the Nanostring Raw data deposited in Gene Expression Omnibus database (GEO- GSE125983) and the sequencing data deposited in Sequence Read Archive (SRA- SRP136344)

Field-specific reporting

Please select the one below that is the best fit for your research. If you are not sure, read the appropriate sections before making your selection.

☒ Life sciences ☐ Behavioural & social sciences ☐ Ecological, evolutionary & environmental sciences

For a reference copy of the document with all sections, see nature.com/documents/nr-reporting-summary-flat.pdf

Life sciences study design

All studies must disclose on these points even when the disclosure is negative.

Sample size	Sample size was estimated based on previous experiments performed in the lab using similar models. This was an exploratory study therefore we could not calculate the sample size as no previous data were available.
Data exclusions	Three mice gavaged with human aspirates were not efficiently colonized and were therefore excluded from the study. Tissue from one recipient mouse receiving CeD donor aspirates presented technical difficulties during embedding and processing, and was dropped out.
Replication	Replication was performed in all experiments and samples when possible. Specific determinations in human duodenal biopsies and aspirates (microbiota, proteolytic activities and mouse colonization) were not replicated due to the low available amount of sample. However several mice per donor were used in most cases.
Randomization	Human aspirates were randomly selected. Mice were sex and age matched.
Blinding	Investigators were blinded to group allocation during data collection and analysis.

Reporting for specific materials, systems and methods

We require information from authors about some types of materials, experimental systems and methods used in many studies. Here, indicate whether each material, system or method listed is relevant to your study. If you are not sure if a list item applies to your research, read the appropriate section before selecting a response.

Materials & experimental systems

n/a	Involved in the study	
☐	☒	Antibodies
☐	☒	Eukaryotic cell lines
☒	☐	Palaeontology
☐	☒	Animals and other organisms
☐	☒	Human research participants
☒	☐	Clinical data

Methods

n/a	Involved in the study	
☒	☐	ChIP-seq
☐	☒	Flow cytometry
☒	☐	MRI-based neuroimaging

Antibodies

Antibodies used	Rabbit anti-human antibody to CD3 (Dako- GA50361-2); Horseradish peroxidase–conjugated anti-mouse IgG (GE Healthcare-NA931); Horseradish peroxidase–conjugated anti-mouse IgA (Abcam- ab97235); Rabbit polyclonal antibody PAR-2 (H-99) (Santa Cruz Biotechnology sc-57797); Alexa Fluor 594 goat anti-rabbit IgG (Life Technologies A11037); CD45-BV421 (30-F11) (BioLegend-103133); CD3ε-PE-dazzle 594 (145-2C11) (BioLegend-100348); CD103-PE (3E7) (BioLegend-121405)
Validation	Dako, GE-Healthcare, Abcam, Santa Cruz Biotechnology, Life Technologies, BioLegend

Eukaryotic cell lines

Policy information about cell lines

Cell line source(s)	Chinese hamster Ovary (CHO) cells, in which NanoLuc luciferase (Nluc) is placed at the PAR-2 N-terminus. Nluc reporter tag was cloned in frame with the human form of PAR-2 cDNA and its stop codon was mutated to insert eYFP tag (Nluc-hPAR2-eYFP).
Authentication	CHO is broadly used and authenticated in the literature. The Vergnolle lab uses these cells routinely for testing cleavage of the external domain of PAR-2. Cells have been described in Mihara K et al. Mol. Pharmacol. 2016
Mycoplasma contamination	All cell lines tested negative for mycoplasma determination

Commonly misidentified lines (See ICLAC register)	N/A

Animals and other organisms

Policy information about studies involving animals; ARRIVE guidelines recommended for reporting animal research

Laboratory animals	Germ-free, clean SPF, and SPF C57BL/6 mice, NOD/DQ8 mice and R38E-PAR2 mice were used. Female and male mice, aged 8 to 12-weeks old mice were used.
Wild animals	N/A
Field-collected samples	No samples collected from the field
Ethics oversight	All experiments were conducted with approval from the McMaster University Animal Care Committee and McMaster Animal Research Ethics Board (AREB) in an amendment to the Animal Utilization Protocol (AUP#170836)

Note that full information on the approval of the study protocol must also be provided in the manuscript.

Human research participants

Policy information about studies involving human research participants

Population characteristics	Adults scheduled for upper gastrointestinal endoscopy suspected of having celiac disease and/or for investigation of anemia, abdominal pain or GERD. Female and male patients were included. Children were not included.
Recruitment	McMaster University Celiac Clinic and McMaster University Digestive Diseases Clinic. Informed consent was obtained from all subjects.
Ethics oversight	The study was approved by the Hamilton Integrated Research Ethics Board (REB # 12-599).

Note that full information on the approval of the study protocol must also be provided in the manuscript.

Flow Cytometry

Plots

Confirm that:

☒ The axis labels state the marker and fluorochrome used (e.g. CD4-FITC).

☒ The axis scales are clearly visible. Include numbers along axes only for bottom left plot of group (a 'group' is an analysis of identical markers).

☒ All plots are contour plots with outliers or pseudocolor plots.

☒ A numerical value for number of cells or percentage (with statistics) is provided.

Methodology

Sample preparation	Single cell suspensions of IELs were stained with fluorochrome-labeled cell-surface antibodies
Instrument	LSR II (BD Biosciences)
Software	FlowJo software (TreeStar, Ashland, OR)
Cell population abundance	The total number of relevant cell populations is found in Supplementary Figure 3c.
Gating strategy	The gating strategy is shown in Supplementary Figure 3d. Live CD45+ cells were gated from single cells, from which the CD3 +CD103+ population was determined. Beads used for determining total cell numbers are shown in the FSC and SSC plot.

☒ Tick this box to confirm that a figure exemplifying the gating strategy is provided in the Supplementary Information.
